# Fownes’ Manual of Chemistry

**Published:** 1885-12

**Authors:** 


### BOOK REVIEWS.

FOWNES' MANUAL OF CHEMISTRY. *New edition. Royal 12mo, 1056 pages. Philadelphia:* LEA BROS. & CO. *Chicago:* JANSEN, MCCLURG & CO.

The original work of Professor Fownes has been edited several times by Mr. Watts, and finally it became so much altered that it amounted to a new book. Shortly before his death, Watts had completed his "Physical and Inorganic Chemistry," and this forms a part of the present edition. It is no exaggeration to say the work thus improved is among the best ever offered to American students. J. H. L.

